# Untouched: understanding the role of touch in obsessive-compulsive disorder

**DOI:** 10.3389/fpsyt.2025.1603464

**Published:** 2025-09-19

**Authors:** Guy Flavian, Gideon Emanuel Anholt, Leehe Peled-Avron

**Affiliations:** ^1^ Psychology Department, Bar-Ilan University, Ramat Gan, Israel; ^2^ Department of Psychology, Ben-Gurion University of the Negev, Beer-Sheva, Israel; ^3^ Gonda Multidisciplinary Brain Research Center, Bar-Ilan University, Ramat Gan, Israel

**Keywords:** OCD, affective touch, ROCD, social, psychotherapy, anhedonia, sensory phenomena

## Abstract

People with Obsessive-Compulsive Disorder (OCD) engage in a wide array of rituals, including both visible behaviors and mental activities, with some of them involving the sense of touch. However, despite the essential role of touch in physical, emotional, and social interactions, the link between touch and OCD has not been thoroughly examined. This review explores the complex and under-investigated relationship between touch and OCD across three domains: (1) sensory phenomena, including “just-right” sensations and tactile over-responsivity; (2) social and interpersonal touch, particularly in the context of avoidance, reassurance-seeking, and comorbidities such as autism spectrum traits; and (3) therapeutic applications, including tactile exposures, mindfulness-based interventions, and somatic practices. We distinguish between discriminatory and affective touch and examine how disruptions in these systems may contribute to symptom expression and maintenance. While empirical research in this area remains limited, we propose a conceptual framework to guide future studies, emphasizing clinical implications for assessment and treatment. Cultural and ethical considerations are also discussed, particularly regarding the use of touch in therapy for individuals with contamination-based OCD.

## 1 Introduction

Obsessive-Compulsive Disorder (OCD) is a psychiatric disorder affecting about 1-2% of the general population and defined by the presence of distressing and intrusive thoughts, impulses, and images (i.e., obsessions), along with repetitive behaviors aimed at reducing anxiety or preventing a feared event (i.e, compulsions) (American psychiatric association, 2013). These symptoms can affect patients’ well-being and interfere with their daily functioning (1). There are numerous shapes to the rituals performed by an individual with OCD. They can manifest as visible behaviors observed by others, like checking if a door is locked, or as mental activities that are not observable, such as repeating a specific phrase in one’s mind (2). The gold standard in assessing the types and severity of OCD symptoms is the Yale-Brown obsessive-compulsive scale (Y-BOCS) (3–5). Some of these compulsions center around the sense of touch and involve physically interacting with objects or people. These touch-based rituals entail an individual repeatedly touching specific objects or individuals in a consistent manner (6). In contrast to initiated touching, some individuals with OCD may exhibit a tendency to refrain from touching, whether it is another person or an object, especially but not limited to, contamination-based OCD (7, 8). In addition to touching rituals, individuals with OCD can experience different sensory phenomena that accompany compulsions, manifesting either prior to or concurrently with the patient’s engagement in repetitive behaviors. Certain occurrences of these phenomena may encompass the tactile sense (9, 10) Apart from within ritual contexts, the sense of touch has an abundance of social, survival, and functional roles (11).

Touch can be broadly divided into two main categories: discriminatory touch and affective touch. Discriminatory touch refers to the ability to detect and distinguish physical characteristics such as texture, pressure, temperature, and spatial location. It is mediated primarily by fast-conducting myelinated A-beta fibers and enables the recognition of objects and environmental cues through tactile exploration. Affective touch, on the other hand, refers to the emotional and social aspects of touch, such as caresses or gentle skin-to-skin contact, often perceived as pleasant or soothing. It is primarily mediated by slower-conducting unmyelinated C-tactile (CT) fibers and plays a critical role in social bonding and emotional regulation (11, 26).

In the physical context, discriminatory touch enables individuals to detect sensations, temperatures, and textures, offering essential information about the surrounding world. Furthermore, touch holds a crucial place in emotional and social interactions, functioning as a fundamental form of communication that conveys feelings of warmth, empathy, and support (11, 12, 26). Within social context, touch was demonstrated to influence social relations and decision making in multiple ways (13, 14). In addition, touch was demonstrated to have a therapeutic effect and may be utilized for mental health and physical therapies to alleviate stress (15, 78)

However, despite the prevalence of compulsions and sensory phenomena in individuals with OCD that involve the sense of touch, no study to date has explored the potential associations between affective and discriminatory touch and OCD. We suggest that investigating this association may yield important insights into how OCD symptoms are influenced by touch, or influence touch as a mechanism that affects distress and functioning. Moreover, understanding the bi-directional influences of OCD and touch may lead to incorporating touch in treatment interventions.

This review researches the question: What is the association between obsessive-compulsive disorder (OCD) and the sense of touch? We approach this question by synthesizing evidence across different domains in which touch may relate to OCD. Our aims are to explore this association in the following key domains:

1. **Sensory Phenomena** – We examine how tactile experiences, including over-responsivity and “not-just-right” sensations, contribute to the onset and maintenance of OCD symptoms.
2. **Social and Interpersonal Touch** – We explore how individuals with OCD engage with social forms of touch, particularly in the context of avoidance, reassurance-seeking, and relational dynamics.
3. **Therapeutic Applications of Touch** – We review how touch-related interventions, including exposure-based therapies and mindfulness-informed techniques, may be leveraged in the treatment of OCD.

To frame this discussion, we distinguish between *discriminatory touch* (which enables detection of physical properties) and *affective touch* (which conveys emotional meaning). We also consider the role of comorbid conditions—such as social anxiety, and autism spectrum traits—in shaping tactile experiences. Through this framework, we aim to integrate current findings, identify critical gaps in the literature, and propose directions for future empirical and clinical research.

## 2 A possible different perception of sensation in OCD

In this section, we focus primarily on sensory phenomena and the role of tactile processing in OCD. It is important to distinguish these phenomena from social or interpersonal touch discussed later in this review. While touch-based compulsions and touch avoidance both involve tactile elements, they arise from different mechanisms: touch-based compulsions (e.g., tapping, touching rituals) are driven by internal urges to reduce distress or reach a “just-right” sensation and often fall under the category of sensory phenomena (3, 9, 10). Touch avoidance, in contrast, is often observed in social contexts and may reflect fear of contamination, anxiety, or discomfort with intimacy. These behaviors are typically avoidant rather than compulsive in nature (7, 8).

### 2.1 Sensory phenomena and over responsiveness

Sensory phenomena (SP) are bodily and mental sensations that can involve muscular-skeletal, visceral, and tactile sensations (16). These sensations can be seen as “not just-right” perceptions that are related to sensory stimuli such as visual, tactile, or auditory cues, sensations of incompleteness, urges, and “just-right” sensations unrelated to any particular stimuli. Examples include - an obsessive thought about a floor not being fully cleaned or a car not parked parallel to the pavement (9, 10). Furthermore, SP has also been shown to be associated with compulsion scores of the Y-BOCS scale (3). Recent clinical studies have offered quantitative insights into the prevalence and nature of touch-related sensory phenomena in OCD. For example, Ferrão et al. (2012) conducted a large-scale study with 1,001 OCD patients and found that 65% reported some form of sensory phenomena (SP). Among those, nearly 80% experienced externally triggered “just-right” sensations, many of which were tactile in nature. Similarly, Summers et al. (2014) demonstrated that tactile and auditory “not-just-right” experiences were significantly associated with obsessive-compulsive symptom severity, particularly in individuals with ordering and symmetry-related compulsions. While these findings are informative, it is important to note that both studies relied on self-report measures, and Summers et al. (2014) was conducted in a non-clinical sample, which may limit generalizability to diagnosed OCD populations.

It is possible that these sensations may derive from early developmental processes that involve sensation, which can contribute to the development of OCD (16). For example, infants with a diminished P50 sensory gating which is a brain measure reflecting inhibitory processes through auditory evoked potentials, had more behavioral difficulties that involve anxiety and attention (17). These difficulties with sensory processing can constitute sensory abnormalities that are observed in people with OCD. There is also some neurological evidence of difficulties with sensory processing in adults with OCD. A study by Rossi et al. (2005) demonstrated reduced sensory gating in people with OCD when compared to a control group. This finding may indicate a difficulty in manipulating sensory information that stems from an elevated resting metabolic activity observed in the orbitofrontal cortex and basal ganglia (18).

Another possibility is that SP in OCD may be explained by sensory over responsiveness (SOR) - a disorder that is defined by an intense adverse reaction to sensory stimuli in one or more sensory modalities (19). When observing the connection between SOR and sensitivity to touch in OCD, SOR to tactile stimulation was demonstrated to produce high levels of anxiety in people with OCD symptoms (20). Although this finding is based solely on self-report questionnaires and a non-clinical population with OCD symptoms, the presence of SOR can exacerbate OC symptoms and interfere with daily functioning - people who are hypersensitive to tactile sensations may find certain textures or physical contact unpleasant, leading them to engage in repetitive rituals to avoid or reduce their discomfort. Another study demonstrated that high SOR is linked to repetitive motor behaviors and insistence on sameness. SOR is present even in children with OCD as approximately one-third of children with OCD demonstrate tactile SOR, while gustatory/olfactory SOR and visual/auditory SOR were reported in only one-fifth of the participants (21). In addition, Dar, Kahn & Carmeli (2012) (22). found correlations between high SOR and everyday experiences that involve oral and tactile stimulations, and childhood ritualism - a common aspect of compulsions in OCD. Their study also indicated an association between oral and tactile hypersensitivity and adult OC symptoms. Taken together, these studies suggest that sensory phenomena are important for gaining a better comprehension of individuals with OCD (9).

### 2.2 The threshold of sensation

Another concept that involves tactile stimulation and OCD is the threshold of sensation (20). The sensitivity of a perceived stimulus can be measured in numerous ways, one of them is to measure when a person experiences a change in the perceived level of a stimulus. This detection threshold is called a just-noticeable difference (JND). When examining the perception of tactile stimuli in OCD patients via sinusoidal mechanical vibrations, people with OCD had no different detection threshold. However, they did demonstrate a difficulty in amplitude discrimination compared with controls (23). While the difference in JND can possibly reflect the “Not Just Right” sensations we mentioned earlier, the lack of a differential detection threshold between OCD and controls suggests no differences in the sensation of a static stimulus. However, different results may have been achieved if a specific OCD SOR subgroup was examined as well. According to their study it is possible that a subtype of OCD that is characterized by more SP experiences exists, particularly in the domains of taste, touch, smell, and vision (19). While the findings offer preliminary insights into the association between sensory over-responsivity and OC symptoms, they are based on self-report data from a non-clinical sample, limiting their generalizability to clinical OCD populations.

### 2.3 Anhedonia & comorbidity with autism traits

There are other disorders and syndromes that demonstrate a different sensitivity to touch and high SOR (24). Some of them have been shown to be strongly linked to OCD, like Gilles de la Tourette Syndrome (GTS) and Autism spectrum disorder (ASD) (25). These comorbidities propose the notion that the pleasantness of touch experienced by people with OCD could potentially be related to its shared components with autistic traits and GTS (25, 26). Phenomenological and family-genetic studies provide evidence for relations between GTS and OCD, predominantly in cases of GTS and tic-related OCD (25). There is a growing body of evidence suggesting that touch is perceived as less pleasant when individuals exhibit autistic traits (26, 27). When compared to typically developed children, children who were diagnosed with autism spectrum disorders (ASD) reported less enjoyment and exhibited more defensiveness while passively touching social and pleasant materials such as skin and fleece (26). This finding is supported by the absence of affective touch awareness and neural activity in the posterior superior temporal sulcus, in people with ASD, that may lead to diminished capability to experience the qualities of affective touch (27). However, the perceived pleasantness of touch is not affected solely by the passive or active presence of touching, as vicarious touch in ASD is also affected. According to Haggarty et al. (2021) (28) Individuals with elevated levels of autistic traits rated vicariously experienced touch as less pleasant in contrast to individuals with lower autistic traits. This aversion from touch, may be explained by increased anhedonia in people with autistic traits (28, 29). A comparable result was observed in individuals with Anorexia Nervosa which is commonly presented with anhedonia (30). Individuals with Anorexia Nervosa perceived affective touch as less pleasurable in comparison to a healthy control group (31). Although no study exists on the perceived pleasantness of touch in OCD, there are some studies that support the presence of anhedonia in OCD, with an estimated 28.3% of people with OCD diagnosed with clinical anhedonia, which also correlated with their Y-BOCS scores (32). This finding may be explained by the importance of the orbitofrontal cortex as a hub for the integration of pleasurable experiences (33). and the reported low volume of the orbitofrontal cortex in OCD patients (34). Although direct research on tactile anhedonia in OCD remains lacking. The current findings should be interpreted as preliminary and hypothesis-generating

To conclude, research suggests that tactile hypersensitivity may have a role as a predisposing factor for OCD and can also exacerbate symptoms in individuals already diagnosed with OCD. Therefore, tactile hypersensitivity should be kept in mind in the assessment and treating people with OCD. Furthermore, it is possible that similar to autism and anorexia, people who experienced anhedonia in OCD may also display a lack of enjoyment from touch. Additionally, this effect may be exacerbated by the presence of tics in OCD, and might even have a more intense effect when a comorbidity between GTS and OCD is displayed. Further research is needed to elucidate the relations between touch OCD and these comorbidities.

## 3 Social touch in OCD

### 3.1 The effects of social touch

Social touch functions not solely as a form of physical contact, but also as a conduit through which individuals convey and receive emotions (35). and have been demonstrated to influence interpersonal behavior in numerous ways (13, 14). For example, Kurzban (2001) revealed that social psychophysical signals such as synchronizing eye gazes, engaging in gentle physical contact, or coordinating rhythmic tapping with each other contribute to an individual willingness to donate to charitable causes via a public goods game. Although this effect was not observed amongst female participants, other studies of nonverbal communication demonstrated this effect among females as well. For example, a study by Crusco & Wetzel (1984) Found that social touch initiated by a waitress increased the amount of tipping she received from both females and males as well (36). Both studies, while influential, rely on behavioral economics and social psychology paradigms in non-clinical populations, limiting their applicability to psychiatric conditions such as OCD.

Affectionate social touch also plays a significant role in establishing and maintaining romantic relationships and defining them. Touch between romantic partners has shown to increase intimacy and strengthen the bond between them (37). Social physical contact with a partner has been linked to immediate enhancements in mood and enduring enhancements in overall psychological welfare. That said, social touch can have negative effects and occasionally be experienced as intrusive, leading to discomfort or stress. This experience has the potential to foster social aversion, particularly among individuals facing difficulties in their interpersonal interactions and social anxiety (Wilhelm et al., 2001). When touched by the experimenter, socially anxious individuals exhibited significantly higher self-reported increases in anxiety, self-consciousness, and embarrassment.

### 3.2 Social touch and mental illness

In the context of mental illness, people who are afflicted with mental conditions, predominantly anxiety and personality disorders, often report challenges in interpersonal interactions that impact both their relationships and their everyday social functioning (39–41). These challenges can range from difficulties in forming and maintaining personal relationships to coping with the stigma associated with mental health conditions, along with avoiding social situations (42). This avoidance naturally reduces social interactions with the outside world and as a result, they engage in fewer instances of social touch (43).

One of the disorders that involves social touch aversion is social phobia, with a study demonstrating that touch initiated by the experimenter on individuals with social anxiety, exhibited significantly higher levels of self-reported anxiety, self-consciousness, and embarrassment in response to the experimenter’s touch (38). In addition, the authors reported that “Socially anxious individuals report that they are less likely to actively pursue touch behaviors in public, private, and intimate situations, and react more negatively to being touched in these situations” (38)P.193-194. Moreover, a reluctance to engage in social touch has been directly associated with social anxiety (44). However, it is important to note that the lack of social touch does not necessarily indicate that a person has social anxiety. People with OCD demonstrate social difficulties as well, such as adjustment difficulties, social inhibition, lack of assertiveness, emotional detachment, and selflessness which can impair their social functioning (45, 46).

### 3.3 Social touch and avoidance

Social anxiety accompanies a wide selection of mental disorders, one of them being OCD, with around twenty to thirty percent of OCD patients diagnosed with a comorbid diagnosis of social phobia (47, 48). In addition, the dwelling on obsessions can lead both children and adults with OCD to frequently enlist the participation of their parents, spouses, and siblings in their day-to-day rituals (1). To confront their obsessions, people with OCD regularly seek reassurance that encourages the feeling of safety, by themselves or via a different person such as a friend or a family member. That form of reassurance is called interpersonal reassurance seeking. A study by Starcevic et al. (2012) found that nearly half of people with OCD seek some form of reassurance from someone, indicating that seeking reassurance is a common strategy for dealing with obsessions. They also discovered that the participants of the study mostly sought reassurance for harm or damage they believe they have caused in the past or will cause in the future. Interpersonal reassurance could also stem from information gathered indirectly, including non-verbal cues in the response (such as the tone of speech and emotional context) (49). This inclusion is aimed to instill a feeling of safety, offer solace, and reduce perceived threats (50).

The social difficulties experienced by people with OCD can also derive from an inflated sense of responsibility and harm obsessions. An inflated sense of responsibility is a belief that one has the power and duty to prevent negative events, and was demonstrated to be related to OC symptoms (51). This burden of over-responsibility may cause one to intervene in events of other people as well (52). The involvement in other people’s events may lead to interpersonal difficulties, by the actions that an individual with a sense of over-responsibility may initiate. It is also possible that because of inflated responsibility, one will distance oneself from social events to prevent the discomfort accompanied by one’s inflated responsibility. The belief one has the power to cause negative events, manifests itself in harm obsessions and compulsions that can cause major discomfort to an individual with OCD (53). In order to refrain from these violent intrusive thoughts, an individual may escape social situations and be very anxious around people. Both elements of OCD may cause a person to reduce the amount of social touch and refrain from touching people and objects that one believes in their power to harm them. The link between inflated responsibility and reduced social touch remains speculative and has not been directly tested in experimental or longitudinal studies.

People with OCD may also become estranged because of certain rituals that they practice to ease their obsessions, which can be seen by other people as concerning and inappropriate. The relatives of people with OCD may even try to hide their family member’s symptoms and prevent them from displaying OC behaviors in public settings (54). Considering all of the social difficulties that an individual with OCD may have to endure, it is understandable that they can report feeling lonely (55) with a recent study in collaboration with the finding that almost three-quarters out of 419 individuals with OCD reported experiencing elevated levels of loneliness (43). It is possible that Touch may serve as a strategy to assess internal states in OCD, although this remains to be empirically validated. The SPIS model (Seeking Proxies for Internal States) proposed by Dar, Lazarov & Liberman (2021). suggests that people with OCD find it difficult to access their internal states, which leads them to seek confirmation of these states via proxies. Touch, may be construed as an internal state with ambiguous meaning that may trigger uncertainty and obsessions (56).

### 3.4 Social touch in ROCD

OCD-related interpersonal challenges can carry to romantic relationships as well, and even be the focus of a certain relationship type of OCD called Relationship Obsessive-Compulsive Disorder (ROCD). These symptoms can encompass a range of intimate relationships, including romantic partnerships, parent-child bonds, mentorships, and religious connections (57, 58). The symptoms that are present in ROCD are repetitive checking of their relationship status, making comparisons to other potential partners, neutralizing (imagining a happy future together), and seeking reassurance. The obsessions linked with ROCD, along with the accompanying compulsive actions, result in significant personal and relational distress (57, 58). Although there is no current data on the use of touching behaviors in ROCD, it is possible that the symptoms in ROCD can affect the use of touch in relationships. For instance, the quantity and manner of touch initiated by a partner may be employed as a mechanism for seeking reassurance. We suggest the connection between ROCD and touch be examined in future research

### 3.5 Social touch and contamination

Yet another factor that is notable in the association between OCD and social touch is fear of contamination. Over half of all individuals with obsessive-compulsive disorder (OCD) present some contamination symptoms. Indeed, that was the primary complaint of 47.6% of patients participating in treatment for OCD (7). A quantitative review of behavioral treatment studies found that contamination and cleaning rituals are among the most frequently reported OCD symptoms, occurring in approximately 50–60% of clinical cases (7). These symptoms are especially relevant to the domain of touch, as they often involve the avoidance of physical contact with perceived contaminants. Additionally, studies on mental contamination—a subtype of contamination OCD—have shown that even without direct physical contact, individuals may experience tactile discomfort, emphasizing the cognitive contribution to touch avoidance (59).

Contamination-based OCD is characterized by avoidance and escape behaviors. These avoidance and escape actions are aimed at warding off feelings of being “contaminated.” Patients often steer clear of substances like bodily fluids, chemicals, and trash. Additionally, they may avoid stimuli that the majority of individuals would consider harmless (8). People with contamination-based OCD believe that the presence of real or imagined infectious, polluted, or hazardous materials could lead to significant harm to their physical or mental well-being, and even pose a social risk (59). While this fear of contamination is evidently linked to reduced touching of objects in one’s surroundings, it can extend to avoidance of social interactions or refraining from touching specific individuals perceived as being contaminated (59). Often, people with contamination-based OCD will even categorize people based on their perceived level of contamination, and those who rank in this hierarchy are actively and adamantly avoided (59). This avoidance possibly reduces a person’s public social touch interactions such as handshakes and hugs, and in severe cases may even disturb intimate relationships by avoiding common romantic gestures of touch such as kissing and sex. It is notable that the feeling of contamination can stem not solely from actual physical touch and there are some people with OCD that demonstrate “mental contamination” such as experiencing a sense of uncleanness without actual physical contact with a contaminating substance, although this form of contamination is not very common (53). One factor that has been examined in OCD based contamination is vicarious touch. A study by Jalal, McNally, Elias, and Ramachandran (2021) (60) demonstrated that people with OCD are influenced by vicarious touch concerning contamination, disgust, and relief. The study found that when people with OCD merely observed the researcher touching a contaminated object, they experienced disgust and distress. Furthermore, when these individuals touched a contaminated object, watching videos of people washing their hands reduced their subjective disgust ratings. An interesting finding was the significant difference between people with OCD and healthy controls; healthy controls were not affected by vicarious touch at all. The severity of OCD played a crucial role, as vicarious touch did not relieve distress in individuals with severe OCD disgust and handwashing urge with moderate OCD severity. However, anxiety relief did not differ between OCD severity.

In conclusion, social touch plays a significant role in our engagement with the environment, yet it can be deliberately avoided in specific mental disorders - most noticeably social anxiety disorder which has high comorbidity rates with OCD. In line with this comorbidity, individuals with OCD demonstrate various behaviorists of social touch avoidance which can harm their relationships and contribute to a feeling of loneliness. It is possible that the ambiguity of touch and its obscure meaning together with the intolerance of uncertainty that is central in OCD also contribute to the difficulties around social touch in OCD. It is also possible that encouraging social touch and mitigating the fear it triggers may be a valuable aspect of OCD treatment. We suggest these ideas be examined in future research.

## 4 Therapeutic effect of touch in OCD

### 4.1 Touch-based interventions

As highlighted in the preceding section, social touch offers numerous advantages. Many studies have tried to utilize the benefits of touch by exploring the connection between touch and therapy. For example, a study by Grennbaum, Lumley, Turner & Melamed (1993) (61) found that the use of touch during a pediatric check at a dentist significantly reduced the fearful and avoidant responses of children. In addition, touch has been shown to decrease pain and fatigue in people suffering from cancer (62). However, the use of touch is not exclusive to physical therapy and physical illness, and some evidence suggests touch can be incorporated into psychological therapy as well (63–65). Touch in psychotherapy involves delicate and non-intrusive physical contact with the intention of cultivating relaxation, mitigating stress, and building trust within the context of therapy. However, the incorporation of touch into therapy remains a subject of significant controversy mostly because of therapy ethics, which focus on possible misinterpretation of touch as a sexual contact (64, 66, 67). One form of using touch in therapy is known as therapeutic touch (TT) - this approach finds its origins in age-old healing traditions across diverse cultures, and is defined as the identification and harmonization of energy. It is rooted in the notion that imbalances and obstructions within the energy field contribute to illness and diminished well-being (65). Although TT is not a prevalent technique in modern psychology, there is some evidence regarding its efficacy in treating anxiety and addressing stress in psychiatric inpatients (68).

Within traditional psychotherapy, there are certain touch applications in various treatment types and counseling, although touch between a patient and a therapist is extremely rare and is typically limited to a handshake (67). Touch, when serving as a means of expressing the therapeutic relationship, is employed for beneficial purposes and can include gestures such as placing an arm on a patient, and embracing a patient (Stenzel & Rupert, 2004). Touch can also be incorporated into therapy within the context of body-oriented psychotherapy, where specific tactile techniques are utilized as components of the therapeutic process (67). It is also possible that touch may have an effect on the therapeutic alliance between a patient and a therapist. McParlin, Cerritelli, Friston and Esteves (2022) (69) suggested that certain elements that form the therapeutic alliance can be enhanced by the use of touch, as touch has a major role in fortifying synchronous relationships and can help in anticipating the mental state of the people that surround us.

Additionally, touch can be used in mindfulness treatments as well. The incorporation of touch in mindfulness therapy interventions has shown notable reductions of depressive symptoms (70). Using mindfulness-based touch therapy that is constructed from awareness of bodily sensations through touch and practicing gentle massage methods Stötter et al. (2013) demonstrated significant reduction of depressive symptoms that were measured by the Hamilton depression rating scale. It is notable however, that Stötter`s study used solely one therapy group and one control group, therefore a comparison between mindfulness based touch therapy and mindfulness therapy or counseling therapy should be a point of interest for future research (70).

### 4.2 The use of touch in OCD treatment

With regards to mental disorders, there is limited to no data about the use of touch or therapeutic touch in traditional psychotherapy treatment. However, there are several studies on the effectiveness of massage therapy. Rapaport et al. (2016) (71) demonstrated that people with generalized anxiety disorder who receive Swedish massage therapy twice a week experienced noteworthy reduction of clinical and self-reported measures of anxiety. It is not clear whether it is due to touch or the general experience of a relaxing environment, since a study by Sherman et al. (2010) found relaxation and thermal rooms to be as effective as massage in reducing symptoms of anxiety. Nevertheless, a review from Rapaport et al. (2018) (15) about massage therapy for psychiatric disorders suggests that inclusion of massage therapy as complementary therapy to psychiatric therapy should be considered. According to their review, collaboration between psychiatrists and massage therapists can result in an approach that leverages the advantages of both disciplines, by combining the therapy of the body and mind.

In the context of OCD, there are both established connections and potential associations between touch and therapeutic interventions. First, as noted in the previous section- people with OCD often experience high levels of loneliness (43). This feeling can possibly be addressed by the use of touch, since being touched has been shown to reduce the perception of loneliness (72). We also noted earlier that OCD frequently co-occurs with social anxiety, which may contribute to social difficulties and the avoidance of interpersonal touch

Social anxiety treatment encourages one to engage more in social activities that might include social touch (73). Although direct empirical data are limited, it is possible that encouraging safe social touch could support improved social functioning and reduced loneliness in individuals with OCD. This warrants further investigation.

Another possible integration of touch in OCD treatment might be in cognitive behavioral treatment (CBT), and most noteworthy - exposure therapy. Touch is used regularly in exposure therapy for OCD in order to help a person confront their fears. For example, in confronting rituals and intrusive thoughts about contamination, an individual is encouraged to touch objects that are believed to be contaminated without performing rituals or neutralizing the contaminants. The implication of touching “untouchable” objects as part of exposure therapy facilitates symptom improvement (74, 75). The use of exposure therapy can also be beneficial for social anxiety symptoms (76). In addition, exposure therapy can include a spouse that can serve as a coach and take an active part in their partner’s treatment (77). Despite the lack of data on the use of social touch in exposure therapy, one can assume that social touch may be used in order to interact with people who are believed to be contaminated, or “untouchable”. In the same manner, the use of social touch in treatment for OCD may target other OC symptoms most noticeably harm obsessions.

The final application of touch in OCD is by providing reassurance. Aside from the avoidance of touch, touch can be a part of the daily rituals performed by people with OCD to provide some reassurance (e.g., touching the wall three times to prevent a disaster) (49). This connection between touch and reassurance has not been examined thoroughly, and the use of social touch as a reassurance method was not examined as well. While there are some studies that investigate the role of interpersonal reassurance in OCD (50), it is noteworthy that no studies have specifically explored the use of touch as a form of interpersonal reassurance. We propose that social touch could play a meaningful role in ROCD-related dynamics; however, empirical studies are needed to investigate this hypothesis. It Is plausible that the frequency and manner in which a partner initiates touch could serve as a strategy for seeking reassurance. However, it is important to acknowledge that this hypothesis requires validation through further research.

To move beyond theoretical speculation, we propose several concrete ways in which touch may be cautiously integrated into established therapeutic frameworks for OCD, particularly under careful clinical supervision and ethical guidelines: In ERP (Exposure and Response Prevention): Physical contact with feared stimuli (e.g., doorknobs, “contaminated” objects) already involves touch-based exposures. Therapists can explicitly frame these tasks as tactile exposures, helping patients attend to the sensory properties (e.g., texture, temperature) without avoidance or ritual. This could also extend to interpersonal touch exposures in cases where patients avoid handshakes or hugs due to contamination fears (74, 75).

In CBT for ROCD or harm obsessions: Therapists may encourage patients to reflect on reassurance-seeking behaviors that involve touch (e.g., hugging to confirm closeness or safety), and help them restructure beliefs around the need for such touch-based confirmation. In behavioral experiments, patients might deliberately refrain from initiating or responding to touch in certain relational contexts to test core beliefs (50, 57).

In Mindfulness-Based Interventions (MBIs): Programs such as mindfulness-based touch therapy (MBTT), which integrate body scans and gentle, guided self-touch (e.g., placing a hand on the chest or abdomen), could be adapted for OCD patients to help reconnect with bodily sensations without judgment. This approach may also support patients who experience touch anhedonia or over-responsivity (70)

These insights suggest that therapist-guided tactile exposures, touch-related cognitive restructuring, and mindfulness-based self-touch practices may offer complementary pathways in OCD treatment — especially for patients with sensory over-responsivity, interpersonal avoidance, or touch anhedonia. Future research should test these approaches systematically.

## 5 Cultural and ethical considerations

The use of touch in therapeutic settings must be approached with cultural and ethical sensitivity. Attitudes toward physical contact vary significantly across cultures, genders, and individual histories, and what may be perceived as supportive in one context could be experienced as intrusive or inappropriate in another (79, 80). This is especially important in OCD presentations involving contamination fears, where tactile exposure exercises can trigger extreme distress. Furthermore, therapeutic touch—even when well-intentioned—raises important ethical concerns regarding consent, boundary maintenance, and the risk of misinterpretation (81). Clinicians should carefully assess each client’s cultural background, personal boundaries, and symptom profile before incorporating any touch-related strategies, and should prioritize verbal consent and collaborative decision-making.

In conclusion, touch has an important role to play in OCD therapy, most noticeably in exposure therapy, and the careful exploration of the role of touch in rituals and avoidance. Similar to other patients, touch within the context of psychological therapy can possibly strengthen the therapeutic alliance between a person with OCD and their therapist. With regards to social functioning, encouragement of social touch can reduce the feeling of loneliness experienced by many people with OCD and help with comorbid social anxiety. Outside of the psychological world, when examining alternative and complementary forms of therapy, some evidence suggests the use of massage might be able to assist in the treatment of OCD. Although no data exists on the inclusion of massage in OCD therapy, people with OCD often experience high levels of stress and anxiety that massage therapy could possibly reduce. These insights suggest that therapist-guided tactile exposures, touch-related cognitive restructuring, and mindfulness-based self-touch practices may offer complementary pathways in OCD treatment — especially for patients with sensory over-responsivity, interpersonal avoidance, or anhedonia. Future research should test these approaches systematically.

## 6 Discussion

In this review, we have explored the connections between OCD and the sense of touch. Despite the limited existing research on this relationship, we have analyzed relevant evidence and pinpointed key areas for a deeper comprehension of touch’s role in OCD and potential directions for future research.

To begin with, people with OCD can experience sensory phenomenon that involve specific tactile and muscle-skeletal experiences. It is possible that these abnormalities in sensory perception may represent a difficulty to manipulate sensory information that stems from a dysfunction in the basal ganglia, which contributes to the formation of OCD. Additionally, individuals with OCD may encounter sensory over-responsiveness, which is a reaction to stimuli that has been demonstrated to be closely linked with anxiety. It is possible that over responsiveness to tactile stimuli can lead to touch aversion as touch can become an aversive stimulus, an aversion that can be accompanied by rituals or avoidance. While there is some evidence about the effects of sensory over responsiveness in OCD, further data is required to determine how sensory over responsiveness that involves specific tactile stimuli, impacts rituals and obsessions. The final notable aspect we observed regarding touch sensitivity in OCD is the absence of pleasure derived from tactile stimuli. Anhedonia is common among individuals with OCD, potentially impacting how they perceive the pleasantness of touch. This finding is enhanced by reported low volume of the orbitofrontal cortex in OCD and comorbidity with autistic traits, both shown to be associated with anhedonia of touch. We propose a more in-depth exploration of anhedonia related to tactile stimuli, as a factor associated with touch avoidance and other rituals involving pleasurable stimuli that might be influenced by anhedonia.

Apart from the abnormalities in sensory perception, touch also holds significance in the relationships and interpersonal connections that individuals with OCD establish and maintain. In addition to the stigma and stress faced by individuals with OCD, they can further strain their interpersonal relationships by involving their siblings, friends, and spouses in their daily rituals. This form of reassurance seeking can be accompanied by an inflated sense of responsibility and harm obsessions that interfere with the interpersonal bonds formed by people with OCD. Such difficulties may explain the high percentage of loneliness in people with OCD and might indicate a lack of social touch, as physical contact was shown to reduce perceptions of loneliness in the general population (Tejada, Dunbar & Montero, 2020). When it comes to romantic relationships and ROCD it is possible that touch is employed as a form of reassurance seeking to determine the relationship status and level of intimacy. ​​Interpersonal reassurance through touch might also be observed in individuals with contamination-based OCD, serving as a method for them to categorize people based on their perceived contamination levels. From another perspective, it is possible that interpersonal touch may enhance the anxiety experienced from obsessions and compulsions in OCD if they are related to the person who touches them in a similar way to that of touching contaminated objects in contamination-based OCD. We propose further research may explore these aspects in depth.

This leads us to propose that the relationship between touch and OCD can be understood סthrough an integrative model comprising three interrelated domains:

1. Sensory touch, which includes abnormalities such as “just-right” sensations, over-responsivity, and tactile anhedonia;
2. Social touch, where avoidance, reassurance-seeking, and interpersonal difficulty manifest;
3. Therapeutic touch, which includes potential applications in exposure therapy, mindfulness, and somatic interventions.

**Figure 1:**
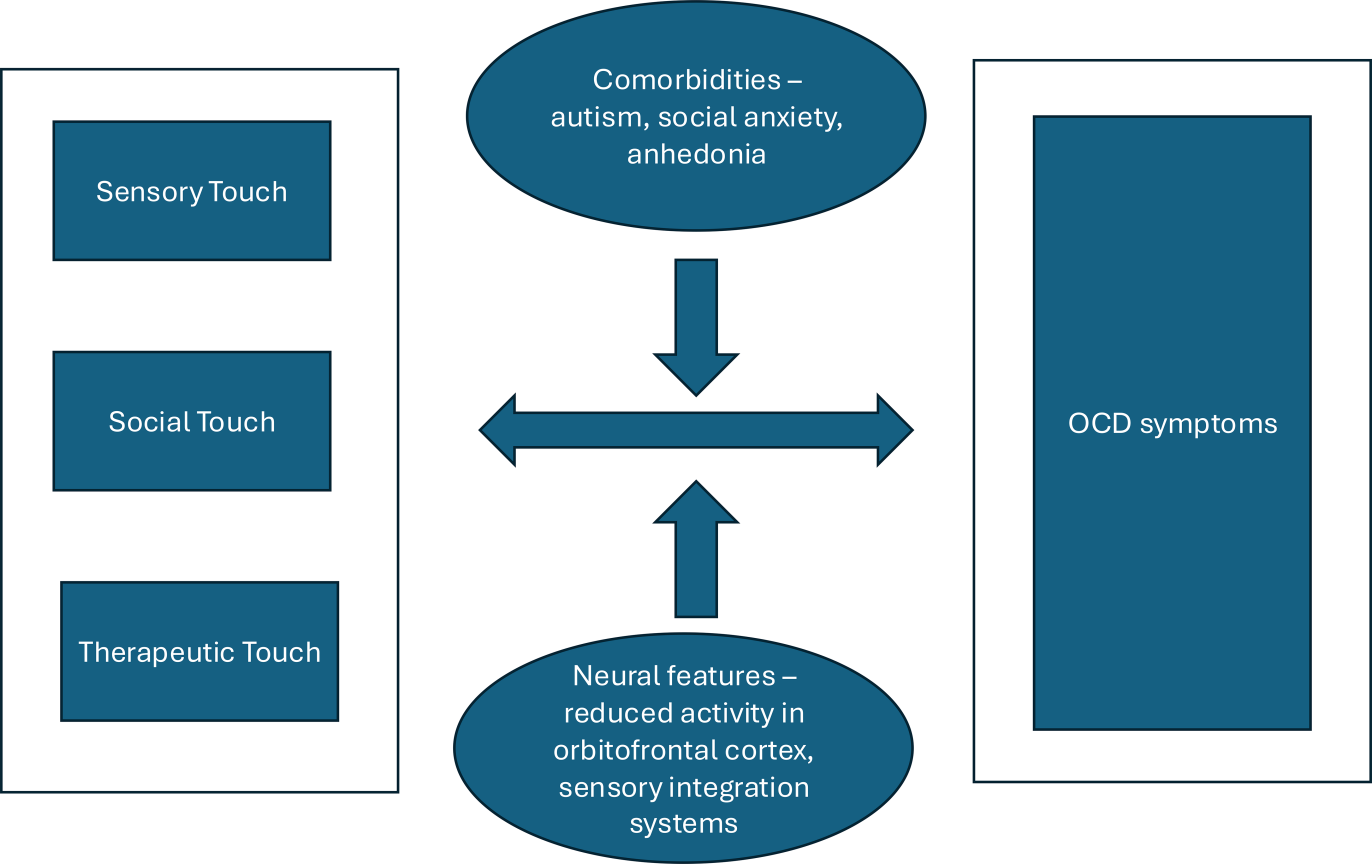
Proposed integrative model comprising three interrelated domains of OCD and touch.

These domains interact within a bidirectional system: OCD symptoms can alter how touch is experienced, while disruptions in tactile processing and social contact may exacerbate obsessive-compulsive patterns. Comorbidities such as autism traits, and social anxiety act as modulators within this system. Neurobiological features, particularly dysfunctions in the orbitofrontal cortex and sensory integration networks, further influence these dynamics. We illustrate this model in **Figure 1**, which may serve as a foundation for hypothesis generation and interdisciplinary research.

## 7 Limitations of the reviewed literature

While this review integrates findings across clinical, psychological, and neuroscientific domains, the strength of the underlying evidence varies substantially. Many studies cited—particularly in the domains of sensory over-responsivity and tactile anhedonia—are based on self-report measures, non-clinical samples, or small exploratory designs. Although these findings offer valuable insights, they should be interpreted cautiously. Moreover, few studies directly examine the causal relationships between touch and OCD symptomatology. Replication in larger, diagnostically verified samples is needed, as is more longitudinal and experimental research to clarify directionality and mechanisms.

While this review seeks to map the association between OCD and the sense of touch, we note that the empirical literature directly addressing this topic remains sparse and methodologically heterogeneous. Due to this lack of cohesive, hypothesis-driven studies, a systematic methodology (e.g., PRISMA) was not applicable. Instead, we adopted a narrative review approach to integrate findings across clinical, cognitive, and neuroscientific domains and to build a conceptual framework for future research. This approach allows us to identify underexplored mechanisms, highlight tensions in the literature (e.g., avoidance *vs*. reassurance-seeking), and raise novel hypotheses. We also attend to ethical and cultural considerations, particularly regarding the therapeutic use of touch in OCD treatment.

In this review, we have also explored the significance of the sense of touch in OCD treatment. Although the use of touch in the psychological treatment of OCD is not prevalent, it may be used to enhance the therapeutic alliance and the synchrony between the patient and the therapist. There is also evidence suggesting that touch can be incorporated into mindfulness therapy, however, further research is needed in order to establish its effectiveness. The use of touch in the treatment of OCD is most notably in aiding OCD patients with touch-related symptoms or comorbid social anxiety in facing social touch situations. Utilizing interpersonal touch in OCD treatment may alleviate the sense of loneliness felt by certain individuals with OCD. Moreover, addressing anhedonia in CBT therapy could potentially decrease these feelings of loneliness, as increased enjoyment from social touch might encourage more frequent engagement in interpersonal interactions.

When it comes to alternative therapy, some evidence suggests that people with OCD may benefit from the inclusion of massage therapy, since massage therapy has shown to reduce anxiety symptoms. Nevertheless, the impact of massage therapy on individuals with OCD remains controversial. We believe this review has shed some light on the possible associations between OCD and the sense of touch and hope it will facilitate future research that will examine the role of touch in the formation of OCD, interpersonal relations, and therapy.

## 8 Summary of key findings and clinical implications

To assist readers in integrating the diverse themes covered in this review, we provide in **Table 1** *a* concise summary table that synthesizes findings across the three primary domains explored: sensory phenomena, social touch, and therapeutic touch. For each domain, we outline the most consistent findings, note the general strength of supporting evidence, and indicate potential implications for clinical assessment and intervention. This overview is not exhaustive but is intended to distill the main points into a practical reference that can guide both clinical practice and future research directions.

**Table 1:** A concise summary table that synthesizes findings across the three primary domains explored in the review.

Domain	Key findings	Evidence type	Clinical implications
Sensory Phenomena	“Just-right” sensations and tactile over-responsivity are common in OCD.	Large-scale surveys, clinical studies	Include tactile SP in assessments; tailor exposure tasks to sensory triggers.
Sensory Phenomena	Altered tactile discrimination and anhedonia reported in some OCD populations.	Small lab-based studies	May inform sensory processing interventions.
Social Touch	Avoidance of interpersonal touch linked to contamination fears and social anxiety.	Clinical samples, experimental tasks	Distinguish between compulsive *vs*. avoidant behaviors in diagnosis.
Social Touch	Reassurance-seeking may involve touch in interpersonal contexts.	Clinical observation, self-report	Address tactile reassurance in therapy to avoid reinforcing compulsions.
Therapeutic Touch	Touch-based exposure can reduce distress in contamination-related OCD.	Case reports, ERP studies	Consider tactile exposure within ERP for relevant cases.
Therapeutic Touch	Mindfulness and somatic awareness may improve tactile comfort and reduce anxiety.	Pilot trials, qualitative studies	Use adjunctively to address tactile discomfort and improve emotional regulation.
